# Costimulation of IL-2 Production through CD28 Is Dependent on the Size of Its Ligand

**DOI:** 10.4049/jimmunol.1500707

**Published:** 2015-10-23

**Authors:** Hong-Sheng Lim, Shaun-Paul Cordoba, Omer Dushek, Jesse Goyette, Alison Taylor, Christopher E. Rudd, P. Anton van der Merwe

**Affiliations:** * Sir William Dunn School of Pathology, University of Oxford, Oxford OX1 3RE, United Kingdom;; † University College London Cancer Institute, University College London, London WC1E 6DD, United Kingdom; and; ‡ Department of Pathology, University of Cambridge, Cambridge CB2 1QP, United Kingdom

## Abstract

Optimal T cell activation typically requires engagement of both the TCR and costimulatory receptors, such as CD28. Engagement of CD28 leads to tyrosine phosphorylation of its cytoplasmic region and recruitment of cytoplasmic signaling proteins. Although the exact mechanism of CD28 signal transduction is unknown, CD28 triggering has similarities to the TCR, which was proposed to use the kinetic-segregation (KS) mechanism. The KS model postulates that, when small receptors engage their ligands within areas of close (∼15 nm) contact in the T cell/APC interface, this facilitates phosphorylation by segregating the engaged receptor/ligand complex from receptor protein tyrosine phosphatases with large ectodomains, such as CD45. To test this hypothesis, we examined the effect of elongating the extracellular region of the CD28 ligand, CD80, on its ability to costimulate IL-2 production by primary T cells. CD80 elongation reduced its costimulatory effect without abrogating CD28 binding. Confocal microscopy revealed that elongated CD80 molecules were less well segregated from CD45 at the T cell/APC interface. T cells expressing CD28 harboring a key tyrosine-170 mutation were less sensitive to CD80 elongation. In summary, the effectiveness of CD28 costimulation is inversely proportional to the dimensions of the CD28-CD80 complex. Small CD28-CD80 complex dimensions are required for optimal costimulation by segregation from large inhibitory tyrosine phosphatases. These results demonstrate the importance of ligand dimensions for optimal costimulation of IL-2 production by T cells and suggest that the KS mechanism contributes to CD28 signaling.

## Introduction

In addition to TCR binding to cognate peptide-MHC (pMHC), the engagement of a second “costimulatory” receptor is usually required for the full activation and differentiation of naive T cells. CD28 was one of the first such costimulatory receptors to be characterized. It binds to ligands B7.1 (CD80) and B7.2 (CD86), which are expressed on professional APCs (1). Numerous functional studies, including experiments on CD28-deficient mice, documented an important role for CD28–ligand engagement in T cell function (2).

CD28 has a single Ig variable–like extracellular domain and a short (41-aa) unstructured cytoplasmic domain. It exists as a disulfide-linked homodimer, although it is functionally monovalent because CD80/CD86 binding to one CD28 sterically prevents CD80/86 binding to the second CD28 in the dimer (3, 4). Signal transduction involves ligand binding–induced phosphorylation of its cytoplasmic domain and recruitment of cytoplasmic signaling proteins (reviewed in Refs. 5, 6). For example, tyrosine phosphorylation of a conserved YMNM motif by Src family kinases (SFKs), such as Lck, leads to its binding to PI3K via the SH2 domain of its p85 subunit (7). Recruited PI3K catalyzes the formation of phosphatidylinositol 3,4-biphosphate and phosphatidylinositol 3,4,5-triphosphate in the membrane. By binding to pleckstrin homology domain–containing proteins, these phospholipids facilitate recruitment and activation of proteins, such as kinases PDK1 and PKB.

Although the signaling pathways activated by CD28 engagement have been extensively characterized, the actual mechanism by which CD80 or CD86 binding induces tyrosine phosphorylation of, and recruitment of proteins to, the CD28 cytoplasmic tail, henceforth referred to as triggering, remains poorly understood. CD28 is a member of a large class of leukocyte cell surface molecules, termed noncatalytic tyrosine phosphorylated receptors (NTRs), which includes the TCR (8). All NTRs signal by phosphorylation of cytoplasmic tyrosine residues by extrinsic tyrosine kinases, typically SFKs, raising the possibility that they share the same triggering mechanism(s). One such mechanism is the kinetic-segregation (KS) model, which was originally postulated to contribute to TCR triggering (9, 10). The KS model postulates that binding of NTRs to their ligands on other cells leads to lateral segregation of the relatively short NTR/ligand complexes from receptor protein tyrosine phosphatases (RPTPs) with bulky extracellular domains, such as CD45 and CD148. In contrast, the SFKs, which are associated with the cytoplasmic leaflet of plasma membrane and lack extracellular domains, are not segregated. The resulting increase in the kinase/phosphatase ratio in the immediate vicinity of the engaged NTR leads to sustained tyrosine phosphorylation of cytoplasmic tyrosine residues.

Although a number of studies provided strong experimental evidence that the KS mechanism contributes to TCR triggering, the role of the KS mechanism in signaling through other NTRs, such as CD28, is less well understood (8, 10). Structural studies, including the crystal structure of the complex between the related receptor CTLA-4 and CD86 (11), suggest that the CD28/ligand complex is likely to be small and span a similar distance (∼15 nm) as the TCR/pMHC complex. Indeed, the CD28/CD80 complex was shown to colocalize with the TCR/pMHC complex in microclusters at the interface between T cells and planar lipid bilayers presenting pMHC and CD80 (12), from which CD45 is excluded (13).

A key prediction of the KS model is that the small size of an NTR/ligand complex is necessary to ensure that engagement occurs in close intermembrane contact areas from which RPTPs with large ectodomains are passively excluded. We showed previously that elongation of the TCR/ligand and other NTR/ligand complexes abrogates NTR signaling (14–16). To test whether the KS mechanism contributes to CD28 signaling, we examined the effect of elongating the extracellular portion of CD80 on its capacity to costimulate IL-2 production by T cells. We found that CD28 costimulation correlates inversely with the size of CD80. Control experiments showed that this was not the result of impaired CD28 binding to elongated CD80 molecules or a mismatch in the dimensions of the TCR/pMHC and CD28/CD80 complexes. Elongated CD80 molecules were less effectively segregated from CD45 at the contact interface, and T cells expressing CD28 with a Y-to-F mutation in the YMNM motif were less affected by elongation of CD80 molecules. Taken together, these results demonstrate that the small size of CD80 is important for its optimal costimulation of IL-2 production via CD28 and support the hypothesis that the KS mechanism contributes to CD28 triggering.

## Materials and Methods

### DNA constructs

Chimeric constructs were generated by PCR-based mutagenesis and verified by dsDNA sequencing (Source BioScience). DNA encoding spacer fragments of human CD2 (amino acids 26–206) or CD4 (amino acids 27–309) with 5′ SacII and 3′ BspEI restriction sites were inserted into the Ig hinge-like region of CD80 extracellular domain between serine 229 and proline 237. DNA encoding GFP with 5′ NotI and 3′ XhoI restriction sites were inserted immediately after the CD80 sequence, with the stop codon removed to create CD80-GFP fusion molecules. The various constructs were cloned into pcDNA3.1^+^/Hygro (Invitrogen) expression vectors.

### Cell lines and mouse cell purification

CHO cell lines expressing H-2K^b^ single-chain dimer (SCD) or IE^K^ were described elsewhere (16, 17). The various CD80 constructs were transfected into the stable CHO cell lines using electroporation, according to the manufacturer’s protocol (Lonza Nucleofactor Kit V for CHO; catalog no. VACA-1003). Cells were sorted for matched expression of CD80 (clone 1G10; Abcam catalog no. AB2555), endogenous hamster ICAM-1 (clone J5-3F9; kindly provided by Vijay Kuchroo, Harvard Medical School), IE^K^ (clone 14-4-4S; BD Biosciences catalog no. 553544), or H2-K^b^ (clone AF688.5; BD Biosciences catalog no. 553570), where relevant, before experiments using a Beckman Coulter MoFlo.

CD4^+^ or CD8^+^ T cells were purified from the spleens using a Dynal Mouse CD4 or CD8 Negative Isolation Kit (Life Technologies catalog no. 11415D), according to the manufacturer’s instructions. DO11.10 CD28-Y170F knock-in mutant mice were kindly provided by Jonathan Green (Washington University School of Medicine).

### T cell stimulation assays and statistical analysis

A total of 5 × 10^4^ CD4^+^ A1 Rag1^−/−^ × CBAc/a T cells or CD8^+^ OTI C57BL/6 T cells was incubated with prepulsed CHO-IE^K^ (with deadbox gene Dby peptide REEALHQFRSGRKPI) or CHO-SCD (OVA_257–264_ peptide SIINFEKL) for 48 h. For *trans*-costimulation assays, 5 × 10^4^ IE^K^-expressing CHO cells were pulsed with 5 μM Dby peptide and incubated with A1 Rag1^−/−^ × CBAc/a T cells at a 1:1 ratio, together with the indicated number of unpulsed, CD80-expressing costimulatory CHO cells. In Fig. 5, TCR stimulation was provided by immobilizing 5 μg/ml anti-CD3 (clone 145-2C11; BioLegend catalog no. 100304) Abs on streptavidin-coated plates (Sigma-Aldrich catalog no. S6940). Supernatants were harvested after 48 h and assayed for IL-2 by ELISA.

To analyze the data from CD28–wild-type (WT) of CD28-Y170F DO11.10 TCR-transgenic mice, the dose-response curves were fitted with a Gaussian function, and the area under the curve was calculated as a measure of IL-2 secretion, referred to as the integrated IL-2 response. In this analysis, data from CD80-CD2 and CD80-CD4 were collated, and the degree of IL-2 secretion impairment as a result of CD80 elongation was quantified by calculating the ratios of elongated CD80/CD80-WT. A paired *t* test analysis was used to determine whether IL-2 secretion from elongated CD80 in CD28-WT T cells was significantly different from T cells with CD28-Y170F.

### CD28-binding assay

Recombinant mouse CD28-Fc chimeric fusion protein (R&D Systems catalog no. 483CD/CF) was titrated in 2-fold dilutions with PBS–1% BSA at the indicated concentrations and shaken with 50 μl protein A–conjugated fluorescent beads (Spherotech; Libertyville catalog no. PAFP-0556-5) for 2 h at room temperature in the dark. The resulting CD28-Fc–coated fluorescent beads were washed, resuspended, and added to 5 × 10^5^ CHO cells expressing the indicated forms of CD80 in 96-well plates. The cells were centrifuged at 1000 rpm at 0°C for 30 min before analysis by flow cytometry.

### Confocal microscopy and image analysis

CD8^+^ OT1 TCR T cells were incubated with 5 μM OVA peptide (pOVA)-SIINFEKL–pulsed or unpulsed CHO-SCD cells expressing the various lengths of CD80-GFP constructs. The cell mixtures were gently dispersed onto a 22-mm round glass slide that was prewashed in 1 M hydrochloric acid and preincubated with poly-l-lysine. Cells on the glass slides were centrifuged at 400 rpm for 2 min at 4°C before incubation at 37°C for 10 min for conjugate formation. The cells were fixed in 4% methanol-free paraformaldehyde for 30 min, permeabilized with 0.1% saponin for 30 min, and stained for CD45 using an Alexa Fluor 647–conjugated anti-human CD45 mAb (clone HI30; BioLegend catalog no. 304020) for 45 min on ice. Cells on the glass slides were washed, mounted, and analyzed immediately.

Images were taken with the Inverted Olympus FV1000 Confocal system at 512 × 512 μm resolution under a 60× UPlanSApo Olympus objective with a numerical aperture of 1.35. Image stacks consisted of 15–20 planes spaced across ∼3–4 μm in total. Three-dimensional reconstruction of the CHO cell–T cell conjugate was performed using Imaris software (Bitplane). Only conjugates whose contact areas were oriented so that they were contained in a rectangular volume for an en face projection were analyzed further.

En face projections of the conjugate interface were analyzed for CD45–Alexa Fluor 647 and CD80–GFP correlations using Image J [PSC colocalization plugin, as detailed in French et al. (18)]. Individual data sets were tested for normality (D’Agostino–Pearson normality test) using Prism 6 (GraphPad Software). One-way ANOVA was used to determine whether the Pearson correlation coefficients were sufficiently different among CD80–WT, CD80–CD2, and CD80–CD4.

## Results

### Elongation of CD80 reduces costimulation of IL-2 production by primary T cells

**FIGURE 1.**
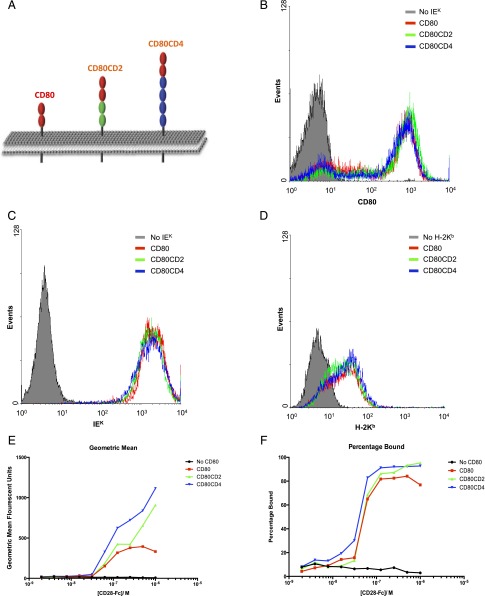
Expression of elongated forms of CD80. (**A**) Schematic view of the elongated versions of CD80 used in this study, with the plasma membrane depicted in gray. The ovals represent IgSF domains. The extracellular portions of human CD2 (two domains, green) or CD4 (four domains, blue) were inserted into the stalk region of mouse CD80 to create the CD80-CD2 and CD80-CD4 chimeras, respectively. CHO cells expressing IE^K^ (**B** and **C**) or H-2K^b^ (**D**) and the indicated forms of CD80 were stained using mAb to CD80 (B), IE^K^ (C) or H-2K^b^ (D). (**E** and **F**) CHO cells expressing different forms of CD80 were incubated with fluorescent beads coated with varying concentrations of CD28-Fc and analyzed by flow cytometry. The geometric mean fluorescent intensity (E) and the proportion of fluorescently labeled cells (F) are shown. Data are representative of three separate experiments.

**FIGURE 2.**
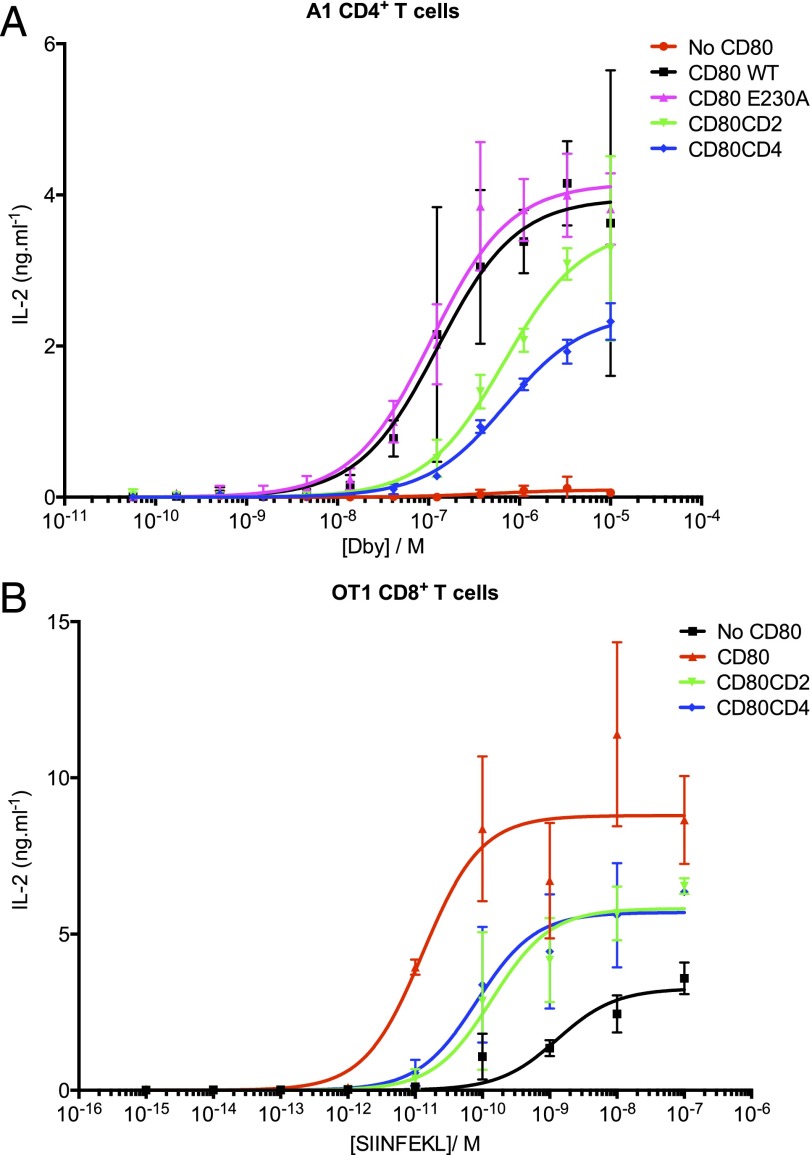
Elongation of CD80 abrogates costimulation. CHO cells expressing IE^K^ (**A**) or H-2K^b^ (**B**) and the indicated forms of CD80 were pulsed with a range of cognate peptide concentrations before incubation with purified CD4^+^ A1 TCR-transgenic T cells (A) or CD8^+^ OT1 TCR-transgenic T cells (B) for 48 h. The supernatants were harvested, and IL-2 was measured by ELISA. The results are representative of at least three separate experiments. Error bars represent the SEM of three replicates.

To investigate the significance of ligand size for CD28 costimulation, we extended the extracellular domain of CD80 by inserting the extracellular portions of human CD2 or CD4 into its stalk region (Fig. 1A). This approach was used previously to elongate several other cell surface molecules, including CD48 (19), MHC class I (16), and MICA (15). Insertion of the spacer domains required the creation of a restriction enzyme site in the sequence encoding the stalk region of CD80, which introduced the E230A mutation. This mutation had no effect on the expression of CD80 or its ability to bind CD28 or costimulate T cells (Fig. 2A). The human CD2 and CD4 inserts are assumed to be functionally inert in our experiments because they bind very weakly, if at all, to the mouse orthologs of their ligands (20, 21).

For functional experiments, the CD80 constructs were expressed in CHO cells expressing appropriate mouse MHC class I (H-2K^b^) or II (IE^K^) molecules. Following sorting by flow cytometry, it was confirmed that these artificial APCs expressed matched levels of CD80 (Fig. 1B), ICAM-1 (data not shown), and, where appropriate, H-2K^b^ or IE^K^ (Fig. 1C, 1D).

To rule out the possibility that elongation of CD80 affected CD28 binding, we compared the binding of CD28-coated beads to CHO cells expressing normal-length or elongated forms of CD80 (Fig. 1E, 1F). CD28-coated beads bound at least as well to CHO cells expressing elongated CD80 as to cells expressing the normal-length CD80, indicating that elongation of CD80 did not impair CD28 binding.

To test whether elongation of CD80 influenced costimulation, we compared the ability of CHO cells expressing normal or elongated forms of CD80, together with cognate pMHC, to stimulate primary CD4 or CD8 T cells from TCR-transgenic mice. Spleen-derived CD4^+^ T cells were purified from A1 TCR-transgenic mice, which recognize the male Ag Dby peptide (REEALHQFRSGRKPI) in the context of IE^K^. CHO-IE^K^ cells not expressing CD80 were unable to stimulate T cells, even when pulsed with high concentrations of Dby peptide (Fig. 2A). In contrast, strong activation was observed by CHO-IE^K^ cells expressing WT CD80, indicating effective costimulation by normal-length CD80. Cells expressing CD80 with the E230A stalk mutation were as effective as cells expressing WT CD80 in mediating costimulation, confirming that the stalk mutation itself had no effect on the function of CD80. In subsequent experiments, CD80 E230A was used as the normal-length form of CD80 for comparison with elongated CD80.

CHO-IE^K^ cells expressing elongated versions of CD80 were significantly less effective at stimulating IL-2 secretion at all concentrations of Dby peptide, indicating that elongation of CD80 abrogates its ability to mediate costimulation (Fig. 2A). Effectiveness at costimulation correlated inversely with the size of CD80, in that CD80-CD4 was less effective at costimulating compared with CD80-CD2.

The effect of CD80 size on costimulation of primary CD8^+^ T cells was also investigated using T cells from OT1 TCR-transgenic mice, which recognize pOVA SIINFEKL in complex with the MHC class I molecule H-2K^b^. For APCs, we used CHO cells expressing the SCD form of H-2K^b^, which consists of the H-2K^b^ H chain fused to β_2_-microglobulin by a flexible glycine/serine linker (16). As observed with CD4^+^ T cells, expression of CD80 dramatically improved IL-2 secretion in response to pulsed peptide; furthermore, the elongated forms of CD80 were less effective that the normal-length form (Fig. 2B). Some IL-2 secretion was observed in the absence of CD80, in line with the previous observations suggesting that CD8 T cells may be less dependent on CD28 engagement than CD4 cells (2).

To exclude any functional effect of interactions between CD80 and PD-L1 on activated T cells (22), we performed similar experiments in the presence of a PD-L1–blocking Ab (clone 10F.9G2) and found no significant difference between control and Ab-treated samples (data not shown).

### Elongation of CD80 impairs CD28 signal transduction

Elongation of CD80 may decrease costimulation of TCR signaling by introducing a mismatch between the dimensions of the CD28/CD80 and the TCR/pMHC complexes. Such a mismatch could disrupt CD28/CD80 and/or TCR/pMHC engagement, because the intermembrane distance cannot be optimal for two interactions that span different distances (19). A mismatch could also disrupt signal integration between TCR and CD28 by preventing their close colocalization with the immunological synapse (15).

**FIGURE 3.**
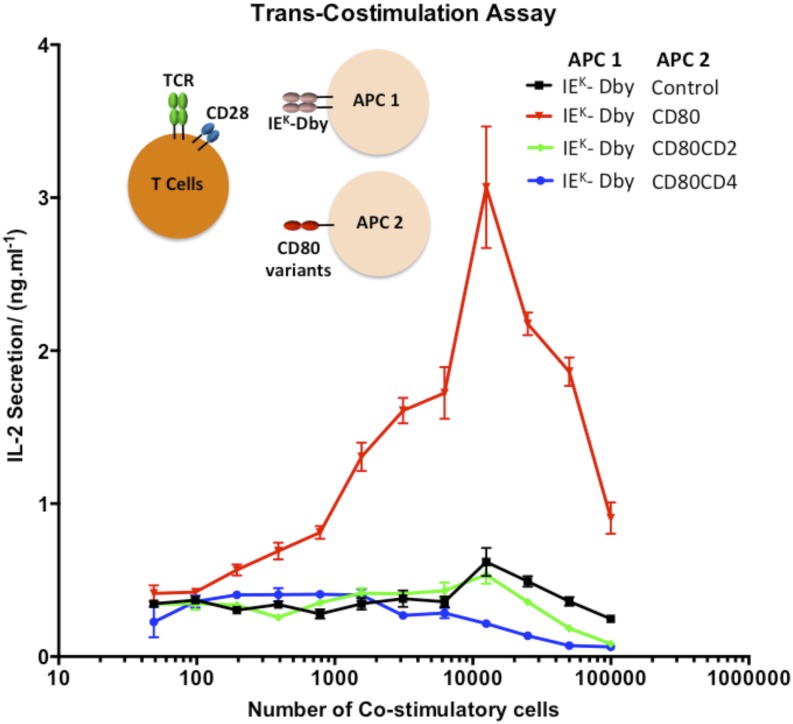
Elongation of CD80 impairs costimulation in *trans.* A total of 5 × 10^4^ CHO cells expressing IE^K^ (APC 1) was pulsed with 5 μM Dby peptide for 1 h and incubated with 5 × 10^4^ primary A1 CD4^+^ T cells and the indicated number of costimulatory CHO cells (second costimulatory APC [APC 2]) for 48 h. The supernatant was harvested, and IL-2 was measured by ELISA. These results are representative of at least three separate experiments. Error bars represent the SEM of three replicates.

To exclude any confounding effect of mismatched dimensions, a *trans*-costimulation assay was set up in which the TCR and CD28 ligands are presented on different APCs (i.e., in *trans*) (Fig. 3). In such a *trans*-costimulation system, TCR and CD28 are engaged within different contact interfaces at the T cell surface, thereby eliminating any potential problem arising from mismatched dimensions.

Dby peptide–pulsed CHO-IE^K^ cells did not stimulate primary A1 CD4^+^ T cells when the second costimulatory APC did not express CD80 (Fig. 3). When the costimulatory APCs expressed normal-length CD80, IL-2 secretion was substantially enhanced, indicating that effective costimulation was being provided in *trans* (Fig. 3). However, when the costimulatory APC presented elongated forms of CD80, little IL-2 secretion was detected, indicating poor costimulation. Because mismatches between the sizes of the TCR/pMHC and CD28/CD80 complexes are irrelevant in this experiment, this suggests that elongated CD80 molecules are poor costimulatory ligands. The decrease in IL-2 secretion observed with higher numbers of costimulatory cells is likely to be the result of overcrowding and/or competition.

### Elongation of CD80 reduces its segregation from CD45 at the contact interface

The KS model postulates that elongation of CD80 inhibits CD28 signaling because the resulting larger CD28/CD80 complex would be less effectively segregated from RPTPs with bulky extracellular domains, such as CD45.

**FIGURE 4.**
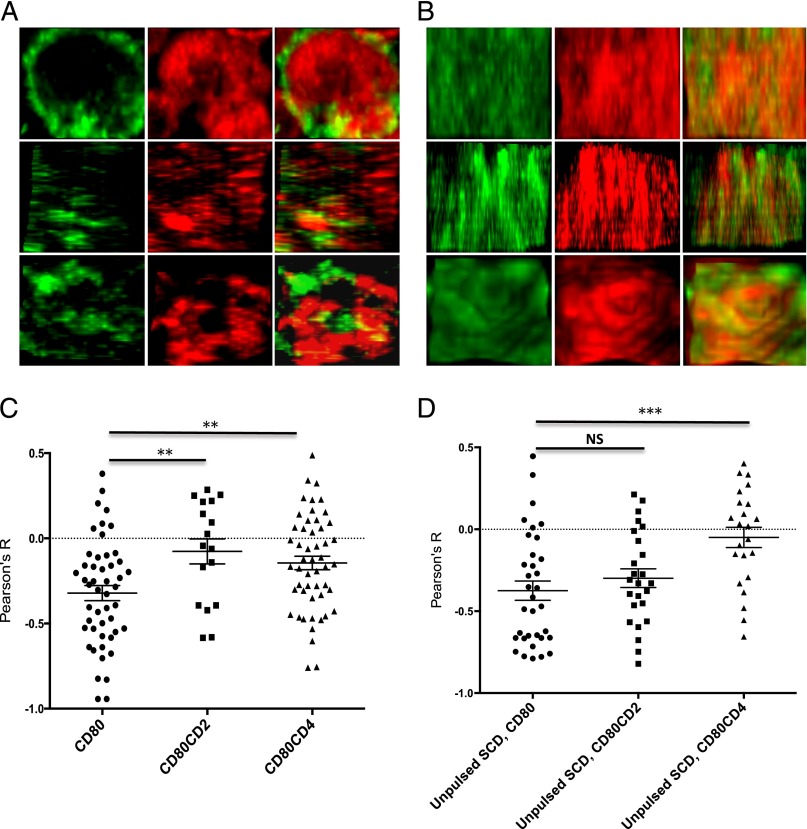
Reduced CD45 segregation from elongated CD80. CHO-SCD cells expressing the indicated form of CD80-GFP were pulsed with pOVA (**A**–**C**) or left unpulsed (**D**) and incubated with primary CD8^+^ OT1 TCR T cells for 10 min. The conjugates were fixed, permeabilized, stained for CD45 using Alexa Fluor 647–conjugated anti-CD45 mAb, and imaged by confocal microscopy. (A) Representative en face views of the interface between pulsed CHO cells expressing CD80-GFP and T cells showing the distribution of CD80 (green) and CD45 (red). (B) Representative en face images of the interface between pulsed CHO cells expressing CD80-CD4-GFP and T cells showing the distribution of CD80 (green) and CD45 (red). Original magnification ×1000. (C and D) Pearson correlation coefficients between CD80 and CD45 in the indicated interfaces were calculated and collated. Horizontal lines with error bars represent mean and SEM. **p < 0.01, ***p < 0.001, one-way ANOVA.

To investigate this, we examined the colocalization of CD80 and CD45 molecules at the T cell–APC contact interface by confocal microscopy. To do this, we fused GFP to the C-terminal end of CD80 molecules to create CD80-GFP, CD80-CD2-GFP, or CD80-CD4-GFP fusion proteins. The various CD80-GFP constructs were transfected into stable CHO cell lines expressing the H2-K^b^ SCD. The APCs were pulsed with 5 μM pOVA and incubated with purified primary CD8^+^ T cells from OT1 TCR-transgenic mice for 10 min before they were permeabilized, stained for CD45 using a fluorescently labeled anti-CD45 mAb, and imaged by confocal microscopy. Fig. 4A shows representative en face views of the interface between a pulsed CHO cell expressing normal length CD80-GFP and a T cell, indicating an apparent segregation of CD80 from CD45. Fig. 4B shows the corresponding en face views between a pulsed CD80-CD4-GFP expressing CHO cells and a T cell.

To quantify the results, we calculated the Pearson’s correlation coefficient (R) between CD80 and CD45 for multiple conjugates. Conjugates with CHO-SCD cells expressing normal length CD80-GFP produced a negative Pearson's (R) value, indicating segregation of CD80 from CD45. When the same analysis was performed using CHO cells expressing elongated forms of CD80, namely CD80-CD2-GFP or CD80-CD4-GFP, the Pearson correlation coefficients were significantly increased, and closer to zero, indicating reduced segregation from CD45 (Fig. 4C). To investigate whether segregation is dependent on TCR engagement, we repeated the experiment with CHO SCD cells that had not been pulsed with pOVA (Fig. 4D). Segregation was still observed between CD45 and WT CD80, and this was significantly reduced with the elongated CD80-CD4 chimera but not with the shorter CD80-CD2 chimera. These results are consistent with the hypothesis that elongated forms of CD80 are less effective at costimulating via CD28 engagement because the elongated CD28/CD80 complex is less well segregated from CD45 at the contact interface.

### CD28 Y170F mutants are less affected by changes in CD80 dimensions

To further investigate the mechanism by which elongation of CD80 abrogates CD28-mediated costimulation, we examined costimulation via a mutant form of CD28 (Y170F) in which the key tyrosine in the YMNM motif has been mutated to phenylalanine and, thus, is resistant to phosphorylation. If the functional effect of elongation of CD80 was the result of reduced tyrosine phosphorylation of CD28, then costimulation via the CD28-Y170F mutant should be less sensitive to CD80 elongation.

**FIGURE 5.**
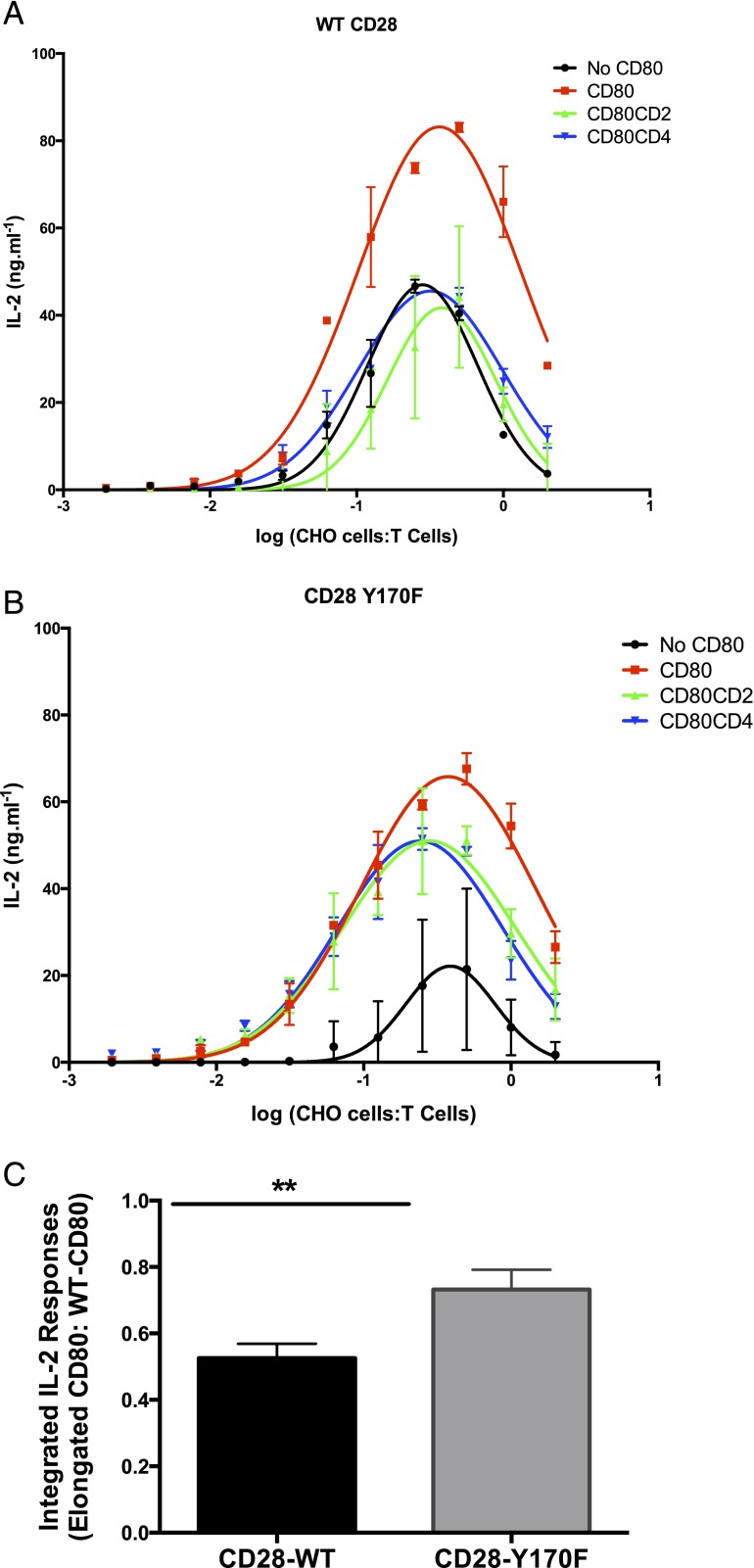
CD28-Y170F mutants are less affected by changes in CD80 dimensions. WT CD4 T cells (**A**) or CD4 T cells harboring the CD28 Y170F mutant (**B**) were stimulated using plate-bound anti-CD3 Abs and incubated with CHO cells expressing no CD80 (control), WT CD80, CD80-CD2, or CD80-CD4. Data are a representative result from at least three experiments. Error bars represent the SEM of three replicates. (**C**) The integrated IL-2 response was calculated by measuring the area under the curves, such as those depicted in (A) and (B). The integrated IL-2 response for elongated CD80 molecules was expressed as a ratio of the response to corresponding normal-length (WT) CD80 molecules within the same experiment. Error bars represent the SEM from five repeat experiments. **p < 0.01, paired *t* test.

TCR engagement was provided by plate-immobilized anti-CD3 Abs, whereas CD28 costimulation was provided by titrating CHO cells expressing normal-length or elongated CD80 molecules. Although CHO cells expressing normal-length CD80 enhanced IL-2 secretion by CD4 T cells expressing WT CD28, CHO cells expressing elongated forms of CD80 were less effective (Fig. 5A). This is consistent with the result above (Figs. 2, 3), confirming that elongation of CD80 disrupts costimulation.

We next examined costimulation of CD28-Y170F–expressing T cells (Fig. 5B). Interestingly, CHO cells expressing CD80 were still able to costimulate these cells, despite the Y170F mutation. This is consistent with many studies reporting that this mutation does not abolish costimulation via CD28 for the production of IL-2 and that signaling motifs other than YMNM contribute to costimulation (23). However, in contrast to costimulation via WT CD28, elongation of CD80 had only modest effects on costimulation via CD28-Y170F mutant (Fig. 5B). To compile and quantify data from multiple experiments, we calculated the integrated IL-2 responses (area under curve) from T cells with WT CD28 or CD28-Y170F when stimulated by WT or elongated CD80 molecules. The integrated IL-2 responses were expressed as the ratio of the response with elongated CD80 versus normal-length CD80 to facilitate comparison. Although elongated CD80 molecules stimulated a lower IL-2 response than normal-length CD80 by both CD28-WT and CD28-Y170F T cells (ratio < 1), the effect of elongation was clearly more pronounced when T cells expressed WT CD28 (Fig. 5C).

## Discussion

In summary, elongation of CD80 inhibits both *cis*- and *trans*-costimulation of IL-2 production by T cells, without impairing CD28 binding, and elongation of CD80 abrogates segregation from CD45 at the contact interface. This suggests that the small size of the CD28/CD80 complex is necessary to ensure its segregation from large inhibitor RPTPs, such as CD45, supporting the hypothesis that the KS mechanism contributes to CD28 triggering.

Additional support for the KS model for CD28 triggering emerged from the analysis of CD28 “superagonist” Abs. These Abs differ from conventional CD28 Abs in their ability to activate T cells without simultaneous TCR ligation (24). Structural and mutational studies showed that superagonist Abs all bind to the membrane-proximal region of CD28 (4, 25). Consequently, when immobilized to surfaces or (via FcRs) to cells, superagonist Abs would be expected to engage CD28 in regions of particularly close membrane apposition, leading to CD45 exclusion and contributing to increased CD28 tyrosine phosphorylation by the KS mechanism (4, 8).

Our finding that T cells with CD28-Y170F mutation were less sensitive to elongation of CD80 than WT T cells supports the notion that elongation of CD80 disrupts tyrosine phosphorylation of the CD28 YMNM motif. The fact that costimulation via CD28-Y170F is still somewhat impaired by CD80 elongation suggests that additional signaling motifs within the CD28 cytoplasmic tail may also be affected by elongation of CD80. One possibility is that one or more of the other tyrosine residues (such as Y188 within the proline-rich PYAP motif) remain susceptible to regulation by large RPTPs and, therefore, are affected by elongation of CD80 (26). Furthermore, the cytoplasmic tail of CD28 was reported to recruit tyrosine kinases, such as Itk and Lck (5). Reduced segregation from RPTPs may also negatively affect phosphorylation of other substrates by these associated tyrosine kinases.

Although our results support a contribution from the KS mechanism to CD28 triggering, they do not exclude other possible mechanisms. The main additional mechanisms that have been proposed for triggering through NTRs, such as CD28 and the TCR, are binding-induced conformational change and binding-induced aggregation (8, 27, 28). Conformational change models postulate that ligand binding somehow induces a conformational change in the cytoplasmic domain. At present, evidence for such a conformational change in the case of CD28 is lacking. Evidence against a specific conformational change as a triggering mechanism is the observation that the ectodomain of CD28 can be replaced by CTLA-4 or the CD80 ectodomain, without compromising signaling (29). Aggregation models require that CD28 binding to its ligands CD80 or CD86 lead to aggregation. The key question is how ligand binding leads to CD28 aggregation. As noted above, the CD28 homodimer is functionally monomeric (3, 4). The fact that CD80 is a noncovalent homodimer raises the possibility that CD80 binding induces dimerization of two CD28 homodimers. Evidence against such a mechanism is the fact that the alternate CD28 ligand CD86 is functionally effective even though it is monomeric, as well as the observation that artificially produced covalent CD80 homodimers are actually less effective than WT CD80 at providing a costimulatory signal (30). Notwithstanding the lack of evidence for a contribution by conformational change or aggregation to CD28 triggering, these mechanisms have not been ruled out, and it is possible that they contribute along with the KS mechanism.

Understanding the mechanisms of NTR triggering is important for the optimal design of chimeric AgRs (CARs), which recently showed considerable promise in tumor immunotherapy (31). CARs typically consist of an extracellular single-chain variable fragment derived from Abs, a cytoplasmic domain derived from the TCR, and T cell costimulatory receptors, including CD28 (31). As such, triggering by CARs is likely to depend on the same processes as these receptors, including the KS mechanism (8). Our finding in this and previous studies (14, 16), that the CD28 and TCR ligand dimensions are critical for triggering, suggests that the design of CARs needs to take this into account. Targeting small cell surface Ags or membrane-proximal regions of larger Ags should enhance CAR triggering by reducing the distance between two opposing cell membranes. Indeed, there have been reports to suggest that this is the case (32, 33).

A limitation of the current study is that it relied on costimulation of IL-2 production by T cells as the functional readout of CD28 engagement. However, CD28 has several cytoplasmic motifs that couple to distinct signaling pathways and are required for different functional effects (23, 34). It follows that not all functional effects of CD28 engagement necessarily depend on the KS mechanism. Further studies are required to dissect the contribution of the KS mechanism to the various signaling pathways and functional effects activated by CD28 engagement.

In conclusion, we showed that elongation of the CD28 ligand CD80 reduced its ability to costimulate IL-2 production by T cells and that this was likely a consequence of reduced signaling through CD28. Elongation also reduced segregation of CD80 molecules from CD45. Finally, CD28 receptors harboring the Y170F mutation were less sensitive to elongation of CD80 dimensions. These results suggest that the KS mechanism contributes to CD28 signaling and support the hypothesis that many NTRs signal by the KS mechanism.
